# The Editor’s Choice for Issue 4, Volume 7

**DOI:** 10.3390/ijns8010021

**Published:** 2022-03-16

**Authors:** David S. Millington

**Affiliations:** Department of Pediatrics, Division of Medical Genetics, Duke University Medical Center, Durham, NC 27709, USA; dmilli@duke.edu

Dear Readers: welcome to the Editor’s Choice for Volume 7, Issue 4 of the *International Journal for Newborn Screening*. This issue comprises over 20 high-quality contributions on a wide range of topics. It was difficult to select just one for special attention. I considered the article by Parad et al. on hospital-based supplemental screening for Duchenne muscular dystrophy [1] worthy of an honorable mention, because it offers an interesting alternative method of recruiting parents and conducting a research study to screen for a condition not yet approved for newborn screening (NBS); other than the one I highlighted [2] in an earlier issue by Kucera et al. [3]. Moreover, two contributions from the Utah NBS program are also worth mentioning. One of these, by Jones et al. [4], addresses a potential framework for the development of a shared data model for standardized electronic data exchange across NBS programs, and the other, by Ruiz-Schultz et al. [5], presents a potential blueprint for a shared bioinformatics platform as more next-generation sequencing (NGS) is adopted for second-tier testing in NBS.

My choice for this issue is the article “Future Perspectives of Newborn Screening for Inborn Errors of Immunity” by Blom et al. [6], for which the excellent review of NBS for SCID by Puck and Gennery in this issue [7] forms a perfect companion piece. NBS for SCID was championed primarily by Dr Rebecca Buckley, who developed hematopoietic stem cell transplantation (HSCT) as the first successful therapy for SCID [8] and Dr Jennifer Puck, who developed the TREC assay as a first-tier NBS test to screen for SCID [9]. NBS for SCID is now widespread, but as the authors point out, there are over 450 recognized inborn errors of immunity (IEI), many of which are treatable by HSCT or other accessible therapies. Methods for expanding the range of immune deficiencies for NBS are discussed. For example, X-linked agammaglobulinemia (XLA) and other severe B-cell deficiencies can be detected by measuring kappa-deleting recombination excision circles (KRECs) in DBS. KRECs can be measured simultaneously with TRECs in a multiplex qPCR-based assay, an approach that has already shown success in pilot studies [10], although the relatively high referral rate for XLA is proving to be a barrier for adoption. Several methods based on tandem mass spectrometry (MS/MS) are discussed, including the targeting of IEI-specific peptides in a multiplex fashion using selected reaction monitoring (SRM). Recently, a panel of eight peptide biomarkers to screen for five molecularly defined IEI, including adenosine deaminase (ADA) deficiency, dedicator of cytokinesis 8 (DOCK8) deficiency, X-linked chronic granulomatous disease (XL-CGD), Wiskott–Aldrich syndrome (WAS), and XLA was reported [11]. The application of molecular technologies to expand NBS for IEIs is extensively discussed, but it would appear that only a targeted approach using next-generation sequencing (NGS) as already used in some NBS programs as a secondary test to the TREC assay [12] would be acceptable as a primary screening option, because the time-sensitive nature of screening for IEI precludes options that result in a delay in reporting results of more than 48 h. Epigenetic immune cell counting is another novel approach that may expand the repertoire of IEIs detectable in newborns [13], but there are problems of scalability and other technical issues that need to be overcome.

Overall, this is a well-written and thorough review of the current literature. It serves to increase our awareness of the technological advances capable of extending the scope NBS for a group of inherited disorders that are devastating unless detected early in life, and for which treatment options are available.
